# Activity of N-coordinated multi-metal-atom active site structures for Pt-free oxygen reduction reaction catalysis: Role of *OH ligands

**DOI:** 10.1038/srep09286

**Published:** 2015-03-19

**Authors:** Edward F. Holby, Christopher D. Taylor

**Affiliations:** 1 Los Alamos National Laboratory, Materials Science and Technology Division, Los Alamos, NM, 87545 USA; 2 DNV GL, Strategic Research & Innovation, Dublin, OH 43017 USA; 3 Fontana Corrosion Center, Dept. of Materials Science & Engineering, The Ohio State University, Columbus, OH 43210 USA

## Abstract

We report calculated oxygen reduction reaction energy pathways on multi-metal-atom structures that have previously been shown to be thermodynamically favorable. We predict that such sites have the ability to spontaneously cleave the O_2_ bond and then will proceed to over-bind reaction intermediates. In particular, the *OH bound state has lower energy than the final 2 H_2_O state at positive potentials. Contrary to traditional surface catalysts, this *OH binding does not poison the multi-metal-atom site but acts as a modifying ligand that will spontaneously form in aqueous environments leading to new active sites that have higher catalytic activities. These *OH bound structures have the highest calculated activity to date.

Pt-free catalysts (PFCs) for the oxygen reduction reaction (ORR) promise to drastically reduce the cost of proton exchange fuel cells (PEFCs) for automotive and stationary power generation^1^. Recent improvements in the activity and durability of such catalysts has rekindled interest in these systems but lack of knowledge of the active site where the ORR occurs has limited the rational, bottom-up design of PFCs^2^,^3^,^4^. Insight regarding the structure of the ORR active site in PFCs would serve to guide synthesis by providing target structures. Maximizing the density of these target structures would lead to greatly improved catalysts, capable of replacing Pt in PEFC cathodes.

Computational studies have proven to be of great use in the rational design of catalyst structures^5^,^6^. A mature formalism for calculating ORR and oxygen evolution reaction (OER) pathways in acidic electrochemical environments has been developed over the previous decade, allowing for calculation of both relative structural stability as well as predicted structure-ORR activity relations^7^,^8^,^9^,^10^. This formalism, and its modifications in which non-canceling entropic, solvation, and zero-point energies are included, has been applied to a number of PFC active site structures^11^,^12^,^13^,^14^,^15^,^16^,^17^ but predicted activities are significantly below what has been observed experimentally. In order to address this gap, different structures must be considered.

By treating active sites as Fe-N defects in a graphene host material, a number of structural motifs have been derived^18^,^19^,^20^. Several properties of these candidate active sites have been elucidated: 1) Fe incorporation is stabilized by nearest-neighbor coordination with N; 2) Fe-N defects are most stable at graphene edges: and 3) Fe-N edge defects are thermodynamically driven to form small clusters, constituting multi-metal-atom sites. These multi-metal-atom PFC sites have been suggested in a number of previous publications^21^,^22^,^23^ but the thermodynamic basis for their existence has only recently been identified.

The multi-metal-atom aspect of the calculated structures can have an impact on the ORR reaction pathway, leading to a dissociative ORR pathway^20^ instead of the associative pathways found in single-metal-atom sites^16^. A more detailed analysis of binding energies of ORR intermediates via use of the computational hydrogen electrode (CHE) methodology and density functional theory (DFT) has been used to calculate the maximum exergonic applied potential, *V_TH_*, i.e., the maximum potential at which no thermodynamic (*TH*) barrier occurs in the calculated reaction pathway between neighboring intermediate states (see, for instance, Ref. 12). Potentials higher than the calculated *V_TH_* have a thermodynamic barrier between intermediate reaction states. The difference between *V_TH_* and the ORR reversible potential (1.23 V) represents a thermodynamic overpotential and thus, the closer *V_TH_* is to the reversible potential, the higher the predicted activity of a given site. This formalism disregards kinetic barriers between intermediate states (as well as solvation and entropic effects) but is useful for comparing relative activities and predicting limiting steps in the ORR reaction pathway. Details of the DFT calculations can be found in the methods section.

The calculated reaction pathway for the multi-metal-atom Fe_2_N_5_ structure is shown in Figure 1. Due to the spontaneous dissociation of O_2_, a dissociative pathway is assumed. It is readily apparent that the reaction pathway at 0 V potential is significantly lower than the ideal pathway, plotted in black, representing overbinding at all ORR intermediate steps. Due to the *OH bound state (reaction coordinate 5) being a lower energy than the ORR final state (2 H_2_O molecules, reaction coordinate 6), the value of *V_TH_* is −0.15 V, which indicates a very inactive ORR catalyst structure. We predict that the *OH bound state would spontaneously form in water bearing aqueous electrolytes and be persistent over a wide range of applied potentials.

In many heterogeneous catalyst systems, overbinding of *OH would be indicative of catalyst poisoning. The persistent *OH would serve to block active surface sites. The three-dimensional nature of the Fe_2_N_5_ site, however, means that even with a persistent *OH ligand, the active site is not sterically hindered from reacting further with an O_2_ molecule. As shown in Figure 2, the *OH is found to bind at the graphene edge host and the bridging N tilts perpendicular to the *OH. This leaves the side opposite the rotated N free to interact with O_2_.

Treating the *OH bound Fe_2_N_5_ site as the thermodynamically stable site in water-bearing electrolytes, we have calculated the reaction pathway energetics as shown in Figure 3. The effect of the *OH ligand and the resulting increased coordination of the Fe atoms drastically increases the calculated activity, leading to a *V_TH_* value of 0.72 V, an increase of 0.87 V versus the *OH free site. The potential determining step in this reaction pathway remains the final protonation step of the *OH intermediate. Additionally, the *OH ligand alters the O_2_ binding such that spontaneous dissociation does not occur and so an associative ORR pathway becomes the more likely pathway.

Due to the multi-metal-atom nature of the predicted stable site, it is interesting to consider the effect of mixed-metal active site structures in similar structures. Such mixed-metal optimization adds an additional parameter that can be used to tune activity in multi-metal-atom sites. It has previously been shown that the combination of Fe and Co can alter catalyst activity in synthesized systems^2^. Exchanging one Fe for a Co atom in the previously considered high activity Fe_2_N_5_(*OH) site leads to an FeCoN_5_(*OH) site structure. This structure and its calculated reaction pathway is shown in Figure 4. This structure has a calculated *V_TH_* value of 0.80 V which is the highest calculated value for any PFC structure to date. The potential determining step in this pathway is the protonation of the *OO intermediate. While direct comparison to experimental data is hindered by mass-transport contributions, this predicted highest activity structure presents an attractive PFC synthesis target.

In summary, stability calculations suggest that spontaneous *OH ligand formation on multi-metal-atom N-coordinated PFC active site structures are thermodynamically likely to be formed during synthesis. Calculated reaction pathways suggest that such sites are significantly more ORR active than single-metal-atom or metal-free sites previously considered in the literature. While it is not possible to definitively say that the presented multi-metal-atom sites are responsible for the high activity found in current state-of-the art PFCs due to a lack of direct observation, these structures are attractive targets for rational design synthesis approaches. Further studies are required in order to include the roles of kinetic barriers between intermediate states, metal atom spin states, solvation of bound intermediates, and zero-point and entropic contributions specific to multi-metal-atom edge sites.

## Methods

Spin-polarized periodic density functional theory (DFT) as implemented in the Vienna *ab initio* Simulation Package (*VASP*)^24^,^25^,^26^,^27^ was used to calculate the relaxed atomic and electronic structure of the candidate active sites as well as intermediate structures for the potential energy surfaces. The GGA method as implemented by Perdew, Burke, and Ernzerhof (PBE-GGA)^28^,^29^ was used to describe the exchange-correlation functional component of the Hamiltonian. An energy cut off of 400 eV was used for the plane-wave basis set. A convergence criteria for the electronic self-consistent loop of 10^−5^ eV was utilized and structures were relaxed until all forces were below 0.02 eV/Å. 8 × 5 carbon pair zig-zag graphene nanoribbons were used as the C-host material for the active sites and vacuum of at least 20 Å was used between periodic ribbons to limit self-interaction. Gamma-point centered 5 × 1 × 1 K-point meshes were used to sample the Brillouin zone with the 1 values corresponding to the two vacuum directions. van der Waals interactions are included in all calculations through the use of the DFT-D2 method of Grimme^30^. A variety of models exist in the literature to include zero-point energy, entropy, and solvation effects. Of particular interest is the work of Lyalin, *et al.*^31^, as it includes the largely balancing effects of all three effects on a B-N surface. Unfortunately, a different reaction pathway than that considered in this work is considered. Szakacs, *et al.*^15^, have interpreted these values for the associative pathway considered here and found the major contribution for the intermediate states to be destabilization of the *O + 2 H^+^ + 2 e^−^ + H_2_O state (Reaction Coordinate 4) by 0.40 eV. Addition of this term does not change the potential determining intermediate states that lead to *V_TH_* for any of the three considered reaction pathways (Reaction Coordinates 5 → 6 and 2 → 3). Instead of adopting this potentially system and exchange-correlation functional dependent methodology, we assume full cancelation of these three effects and report pathways based solely on calculated binding energies from internal energies. This cancelation assumption has previously been shown a fair approximation for ORR intermediates on Pt surfaces^7^,^32^. Further justification of cancelation of ZPE, entropy, and solvation effects is the focus of future work.

## Author Contributions

**Figure 1:**
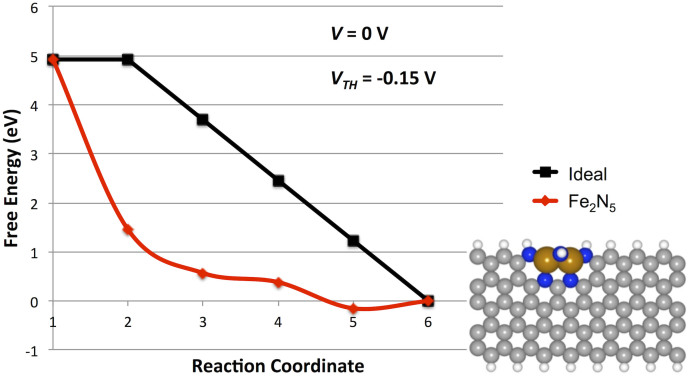
Calculated ORR dissociative pathway potential energy surface for Fe_2_N_5_ edge structure (inset). Reaction coordinates correspond to the given systems: (1) O_2_ +4 H^+^ + 4 e^−^, (2) *O + *O + 4 H^+^ + 4 e^−^, (3) *O + *OH + 3 H^+^ + 3 e^−^, (4) *O + 2 H^+^ + 2 e^−^ + H_2_O, (5) *OH + H^+^ + e^−^ + H_2_O, (6) * + 2 H_2_O.

**Figure 2:**
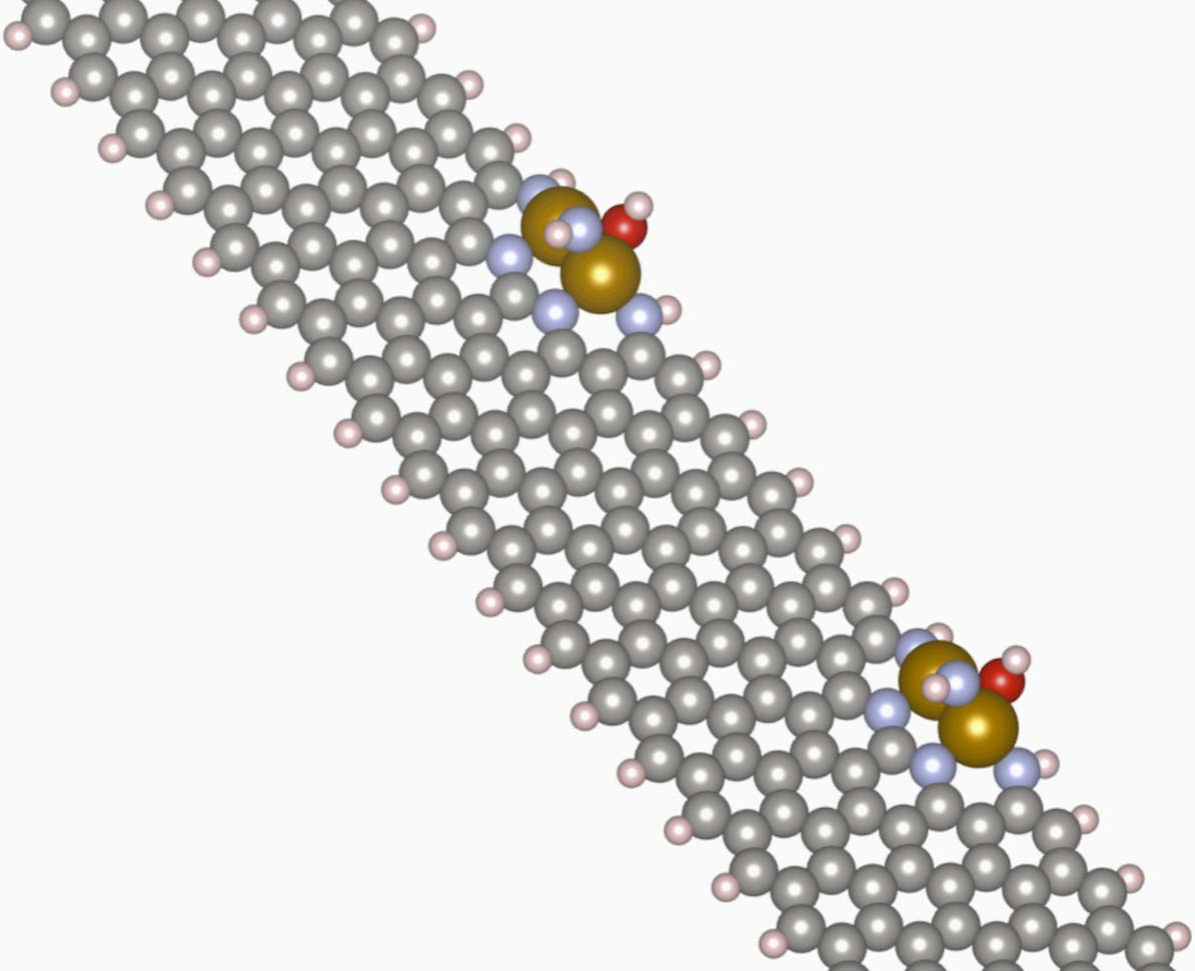
Relaxed Fe_2_N_5_(*OH) edge structure showing locally minimized active site structure with bound *OH. Note that the site is not fully blocked, as is likely to occur in a typical surface catalyst, since the opposite side of the active site is still accessible to reactants.

**Figure 3:**
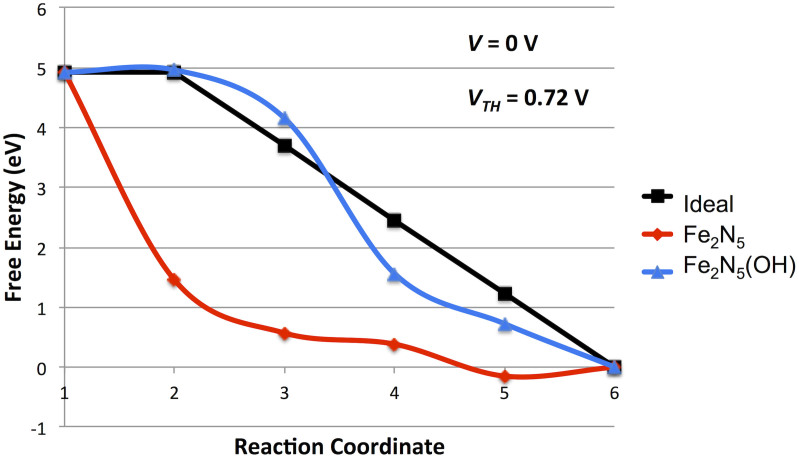
Calculated ORR potential energy surfaces with and without *OH. Reaction coordinates for the Fe_2_N_5_ structure's dissociative pathway correspond to those of Figure 1. Reaction coordinates for the *OH bound structure correspond to the given systems: (1) O_2_ +4 H^+^ + 4 e^−^, (2) *OO + 4 H^+^ + 4 e^−^, (3) *OOH + 3 H^+^ + 3 e^−^, (4) *O + 2 H^+^ + 2 e^−^ + H_2_O, (5) *OH + H^+^ + e^−^ + H_2_O, (6) * + 2 H_2_O.

**Figure 4:**
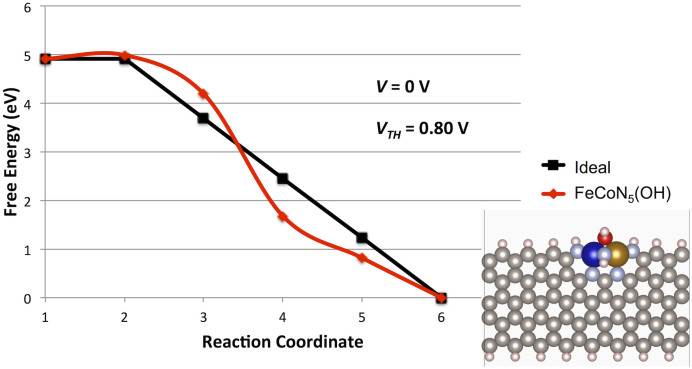
Calculated ORR potential energy surface for mixed-multi-metal atom FeCoN_5_ structure with *OH ligand. Reaction correspond to the given systems: (1) O_2_ +4 H^+^ + 4 e^−^, (2) *OO + 4 H^+^ + 4 e^−^, (3) *OOH + 3 H^+^ + 3 e^−^, (4) *O + 2 H^+^ + 2 e^−^ + H_2_O, (5) *OH + H^+^ + e^−^ + H_2_O, (6) * + 2 H_2_O. For all intermediates, adsorption on the Fe atom was lower energy than on the Co atom. The adsorbate free structure is shown as an inset.

C.T. and E.H. prepared the main manuscript. E.H. performed the DFT calculations and prepared Figures 1–4. All authors contributed to data analysis and reviewed the manuscript.
